# Patient and public engagement in research and health system decision making: A systematic review of evaluation tools

**DOI:** 10.1111/hex.12804

**Published:** 2018-07-30

**Authors:** Antoine Boivin, Audrey L'Espérance, François‐Pierre Gauvin, Vincent Dumez, Ann C. Macaulay, Pascale Lehoux, Julia Abelson

**Affiliations:** ^1^ University of Montreal Hospital Research Center (CRCHUM) Montreal QC Canada; ^2^ Department of family medicine University of Montreal Montreal QC Canada; ^3^ Center of Excellence on Partnership with Patients and the Public (CEPPP) Montreal QC Canada; ^4^ Department of health management, evaluation and policy Ecole de santé publique de l'Université de Montréal Montreal QC Canada; ^5^ McMaster Health Forum McMaster University Hamilton ON Canada; ^6^ Direction Collaboration et Partenariat Patient Faculté de Médecine Université de Montréal Montreal QC Canada; ^7^ Participatory Research at McGill Department of Family Medicine McGill University Montreal QC Canada; ^8^ Centre for Health Economics and Policy Analysis (CHEPA) McMaster University Hamilton ON Canada

**Keywords:** evaluation instruments, patient and public engagement, quality improvement, research, systematic review

## Abstract

**Background:**

Patient and public engagement is growing, but evaluative efforts remain limited. Reviews looking at evaluation tools for patient engagement in individual decision making do exist, but no similar articles in research and health systems have been published.

**Objective:**

Systematically review and appraise evaluation tools for patient and public engagement in research and health system decision making.

**Methods:**

We searched literature published between January 1980 and February 2016. Electronic databases (Ovid MEDLINE, Embase, Cochrane Database of Systematic Reviews, CINAHL and PsycINFO) were consulted, as well as grey literature obtained through Google, subject‐matter experts, social media and engagement organization websites. Two independent reviewers appraised the evaluation tools based on 4 assessment criteria: scientific rigour, patient and public perspective, comprehensiveness and usability.

**Results:**

In total, 10 663 unique references were identified, 27 were included. Most of these tools were developed in the last decade and were designed to support improvement of engagement activities. Only 11% of tools were explicitly based on a literature review, and just 7% were tested for reliability. Patients and members of the public were involved in designing 56% of the tools, mainly in the piloting stage, and 18.5% of tools were designed to report evaluation results to patients and the public.

**Conclusion:**

A growing number of evaluation tools are available to support patient and public engagement in research and health system decision making. However, the scientific rigour with which such evaluation tools are developed could be improved, as well as the level of patient and public engagement in their design and reporting.

## 1 INTRODUCTION

In the past decade, there have been rapid developments in patient and public engagement^1^, ^2^ as illustrated by a tenfold increase in the number of articles published annually on the subject.^3^ As the commitment to patient and public engagement has grown, so too has the call for robust evaluations.^4^, ^5^ Without adequate evaluation tools in place, it is difficult to ensure the integrity of engagement principles and practices, assess the outcomes of engagement, learn from current practices and demonstrate accountability for public investments.

Evaluative efforts on patient and public engagement have expanded recently, with the development of evaluation principles and frameworks^4^, ^6^, ^7^, ^8^, ^9^, ^10^ and an increasing number of published evaluations.^6^, ^11^, ^12^, ^13^, ^14^, ^15^, ^16^, ^17^, ^18^, ^19^, ^20^ The development of structured evaluation tools has been slower, and mostly performed through unpublished, project‐specific instruments, thus limiting the potential for comparison and mutual learning across engagement projects. Systematic reviews of evaluation instruments have been conducted for patient engagement in individual health‐care decision making,^21^, ^22^, ^23^ but need to be expanded to other domains of patient engagement, including research and health system decision making.

## 2 OBJECTIVE

The aim of this study was to systematically review and appraise existing evaluation instruments for patient and public engagement in research and health system decision making.

This work was conducted as part of the Canadian Strategy for Patient‐Oriented Research (SPOR), which promotes patient engagement in research and health system transformation, hence our focus on these 2 engagement domains.^24^ As part of the SPOR strategy, methodological SUPPORT Units (Support for People and Patient‐Oriented Research and Trials) in each province are mandated with strengthening patient and public engagement. We also conducted a systematic assessment of identified evaluation tool to characterize their main strengths and weaknesses, guide engagement practitioners’ choice of specific instruments and orient the future development of such tools.

## 3 METHODS

### 3.1 Definitions and scope

This systematic review was based on CIHR's definition of “patient engagement” as a meaningful and active collaboration in the governance, priority setting, and conduct of research, as well as in knowledge translation.^24^ The “public” includes any “people who bring the collective voice of specific, affected communities.”^24^ “Evaluation” refers to “the systematic acquisition and assessment of information to provide useful feedback about some object.”^25^ Within the context of this systematic review, evaluations could focus on the context, process or impacts of patient and public engagement.^6^ “Evaluation tool” refers to any instrument that can help to systematically acquire and assess information about patient and public engagement activities. This may include questionnaires, scales, interview guides or observation grids for use by engagement participants, organizational sponsors or external evaluators.

### 3.2 Review method

We chose a critical interpretive synthesis method to guide our review. Critical interpretive synthesis allows for the conceptual translation of quantitative and qualitative studies, as well as non‐empirical papers.^26^ This approach is particularly well suited to the synthesis of diverse types of literature, such as quantitative and qualitative, published and grey, and health and social sciences, for which the phenomena of interest, populations, interventions and outcomes vary and may not be well‐defined.

### 3.3 Search strategy

We searched for literature published between January 1980 and February 2016 in the Ovid MEDLINE, Embase, Cochrane database of systematic reviews, CINAHL and PsycINFO databases, without any language restrictions. An information specialist was consulted to help develop, update and execute specific search strategies and bibliographic queries for each database (Table 1).

**Table 1:** Search terms

Public	Patient	Engagement	Evaluation	Tool	Health
Public Citizen Consumer	Patient Service user	Engag* Involv* Participat* Consultat* Partner*	Evaluat* Assess* Measur* Effectiveness Process Quality assessment	Tool Instrument Questionnaire Scale Grid Guide Framework	Health (care) policy Health (care) programme Health (care) services Health (care) research

Concepts listed in columns (eg, engagement, evaluation) were combined with « AND » (with the exception of the concepts « patients » or « public »), while search items listed under each columns (eg, scale, grid) were combined with « OR ». *A “wildcard” in search strategy (ex. part* can refer to participation, participate, participated, etc).

Five additional strategies were used to complement the electronic database search: (i) contacting authors of published evaluation reports; (ii) hand‐searching bibliographies from retrieved articles; (iii) searching for unpublished evaluation tools in Google, Google Scholar and the websites of select Canadian and international organizations (eg, the Patient Engagement Resource Hub of the Canadian Foundation for Healthcare Improvement and the INVOLVE library in the United Kingdom); (iv) soliciting recommendations from Canadian and international experts and networks; and (v) soliciting the input of a larger audience through social media.

### 3.4 Inclusion and exclusion criteria

We included original and review articles on evaluations of patient and public engagement in research and health system decision making that included evaluation tools, and background papers offering critical discussions of key evaluation tools that pertain to patient and public engagement in research and health system decision making.

Documents were excluded if they focused strictly on patient and public communication; patient engagement in individual health‐care decisions; mechanisms to engage patients and the public as research subjects; theoretical and/or conceptual frameworks that were not linked to an evaluation tool; and evaluation tools not used in the context of research and health system decision making.

### 3.5 Data extraction

All search results were transferred to a reference database and duplicates were removed. The titles and abstracts were screened by 2 members of the research team before retrieving the full‐text versions of the references included. Discrepancies between reviewers were resolved by consensus. In total, 4 reviewers were involved in the inclusion process, and 1 reviewed all citations, abstracts and articles.

Two research team members independently reviewed and summarized the peer‐reviewed articles and grey literature using a standardized extraction sheet, compiling the following information: authors; year of publication; main stated purpose; and the name, dimensions and domains, development procedure, theoretical/conceptual foundation and psychometric properties of the evaluation tool. Tool developers who did not publish the tools but only the process or use of their tool were contacted.

### 3.6 Tool assessment

Assessment criteria were developed in collaboration with the evaluation tools’ intended users. Each evaluation tool was assessed based on 4 criteria:

1. Scientific rigour: Was the development of the evaluation tool scientifically rigourous and based on existing evidence on patient and public engagement (eg, based on a literature review in at least 2 databases, assessed for reliability and validity)?
2. Patient and public perspective: Does the evaluation tool take into account the views of patients and the public (both in its development, use and reporting)?
3. Comprehensiveness: Is the tool comprehensive in evaluating the context, process, outcomes and impacts of patient and public engagement?
4. Usability: Is the evaluation tool easy to use (eg, availability and readability level)?

Two members of the research team independently appraised the evaluation tools using the assessment grid and its 5‐point rating system. (See Data S1 for the complete assessment grid.) The evaluation tools’ readability level was assessed using the Flesch Reading Ease test (score of 70 or more, or 7th‐grade level).

### 3.7 Integrated knowledge translation

Evaluation tool users, including patients and members of the public with engagement experience, were involved in all stages of the project, using an integrated knowledge translation approach.^27^ One patient partner was involved in the research team and participated in study design, governance, interpretation and knowledge translation. A steering committee composed of representatives from all sponsoring SUPPORT Units (including patient and public engagement practitioners) met 6 times during the project to review the design, assessment criteria, preliminary findings and knowledge translation strategy. A 1‐day face‐to‐face consensus meeting was held mid‐project to review preliminary findings, discuss assessment criteria and develop the knowledge translation strategy. Thirty‐one people participated in the consensus meeting, half of whom were patients and members of the public with engagement experience, along with researchers, patient engagement practitioners and representatives from national research and health‐care organizations (see Acknowledgement section). An open‐access online evaluation toolkit (http://www.ceppp.ca/en/our-projects/evaluation-toolkit/) including a description of all included evaluation tools was published upon project completion to facilitate dissemination and uptake. End users’ engagement modified the evaluation criteria (eg, adding a focus on usability) and dissemination strategy (eg, open‐access online dissemination).

## 4 RESULTS

**Figure 1:**
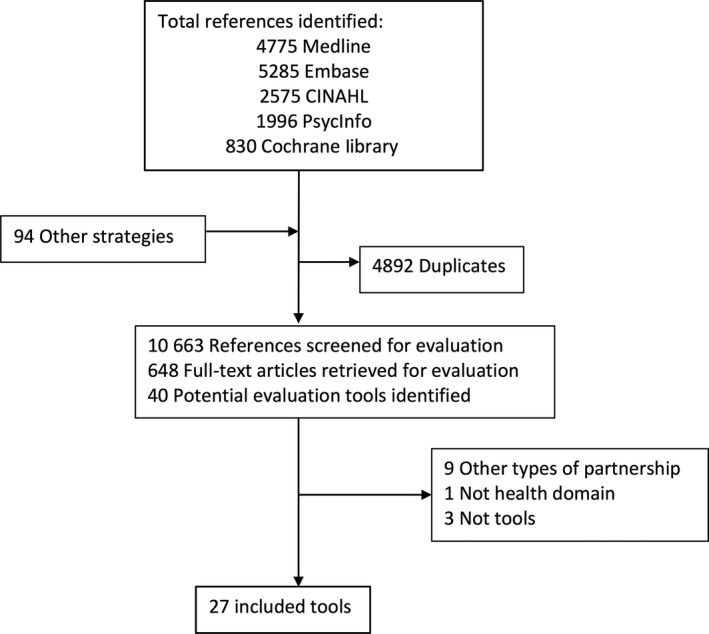
Number of references identified through the stages of the systematic review

After removing duplicates, 10 663 unique references were identified and 648 full‐text articles were reviewed (Figure 1). Forty potential evaluation tools were identified. Thirteen of those were excluded because they were not specific to patient and public engagement (n = 9), did not relate to research and health system decision making (n = 1) or did not actually include an evaluation tool (n = 3). Few evaluation tools’ developers responded to requests for additional information and unpublished tools.

### 4.1 Description of evaluation tools

Twenty‐seven patient and public engagement evaluation tools met the inclusion criteria (full references for those included are found in Table S1). Of the tools included, 14 are specific to research, 11 focus on health system decision making and 2 can be applied to both domains. All of the tools were published in English and the majority of them (85%) were developed after 2005. Most were developed in the United Kingdom (n = 10), Canada (n = 8) and the United States (n = 7). Self‐administered questionnaires and surveys were the most common type of tool identified (n = 15). T he stated purpose behind most of the tools was the improvement of patient and public engagement activities. Table 2 describes the included evaluation tools.

**Table 2:** Description of evaluation tools

Tool	Authors	Country	Year	Type	Objectives
A resource toolkit for engaging patient and families at the planning table	Alberta Health Services Engagement and Patient Experience Department	Canada	2014	Survey and Scale	Two instruments meant to (i) routinely evaluate team collaboration skills and assess your growth and (ii) assess how your team is doing at encouraging participation and collaboration at your meetings
An evaluation of in‐person and online engagement in central Newfoundland	Wilton, Peter, et al	Canada	2015	Surveys	To evaluate the use of in‐person focus groups and online engagement within the context of a large public engagement initiative conducted in rural Newfoundland
Checklist for attitudes for patients and families as advisors	Institute for patient and family‐centred care	USA	2010	Checklist	To explore attitudes about patient and family involvement as advisors and/or members of improvement and redesign teams
Community engagement and participation in research measure	Goodman, Melody S., et al	USA	2017	Questionnaire	To quantitatively measure community engagement participation in health research, based on the principles of community‐engaged research
Community Engagement in Research Index (CERI)	Khodyakov, Dmitry, et al	USA	2013	Index	To offer a multidimensional view of community engagement in the research process
Engaging patients as partners in practice improvement	Willard‐Grace, Rachel, et al	USA	2016	Questionnaire	To assess current strategies, attitudes, facilitators, and barriers towards engaging patients in practice improvement efforts
Evaluating the participatory process in a community‐based heart health project	Naylor, Patti‐Jean, et al	Canada	2002	Rating scales and Sextagram	To evaluate the community‐based participatory process as an indicator of success
Health Democracy Index	Souliotis, Kyriakos, et al	Greece	2016	Index	To assess Patient Association participation in health policy decision making
Involvement portfolio	NHS Forum Service User and Carer Working Group	UK	2015	Portfolio	To record and provide evidence of involvement activities
Kroutil checklist	Kroutil, Larry A., and Eugenia Eng.	USA	1989	Checklist	To review and score project plans to assess planners’ intentions to elicit community participation along 5 dimensions: who participates, in what activities, and through which process or how, given the project characteristics, and the conditions in the task environment
Measuring Organisational Readiness for patient Engagement (MORE)	Oostendorp, L. J., Durand, M. A., Lloyd, A., & Elwyn, G	UK	2015	Scale	To enable a timely assessment of organizational readiness to support a tailored implementation strategy
Organisational Self‐Assessment and Planning (OSAP) Tool	National Resource Centre for Consumer Participation in Health	Australia	2003	Questionnaire	To assist organizations in improving consumer and community participation policies and practice; to identify opportunities for participation; to assess and develop organizations’ commitment and capacity to involve and support consumers and communities in planning, implementation and evaluation activities
Partnership Assessment In community‐based Research (PAIR)	Arora, Prerna G., et al	USA	2015	Questionnaire	To measure important dimensions of the relationship between researchers and community members collaborating on community‐based programming and research
Patients as partners in research surveys	Maybee, Alies et Brian Clark for Patients Canada	Canada	2016	Surveys	To understand the actual experience of researchers when they partner with patients and caregivers on a project where the patients and/or caregivers are members of the research team and to identify behaviours that support productive partnerships
PCORI engagement activity inventory	Patient‐Centered Outcomes Research Institute	USA	2016	Survey	(i) To capture researchers’ experience with patient and other stakeholders engagement in research, (ii) to describe the role of patients and other health‐care stakeholders in research projects and (iii) to describe engagement in research from the researcher point of view
PEI Engagement Toolkit	Health Prince Edward Island	Canada	2016	Checklists, Scales and Questionnaires	To evaluate (i) public or patient engagement process, (ii) team collaboration skills, (iii) how effective engagement meetings are with patients, families, and/or staff, (iv) if guiding principles for effective and meaningful public/patient engagement were met, (v) to gather feedback from participants and staff about their experience
Public and Patient Engagement Evaluation Tool (PPEET)	Julia Abelson and the PPE Research‐Practice Collaborative	Canada	2015	Questionnaires	Consists of (i) an Organization questionnaire to assess the organization's capacity for and culture of public and patient engagement; (ii) a Participant questionnaire to obtain participants’ assessments of key features of the engagement activity that they have participated in, and (iii) a Project questionnaire to assess the planning, execution and impact of the engagement activity after it has been completed
Public Involvement Impact Assessment Framework (PiiAF)	Popay, J., M. Collins, and the PiiAF Study Group	UK	2014	Framework	To help researchers assess the impacts of involving members of the public in health and social care research
Quality Involvement Questionnaire	Morrow, Elizabeth, et al	UK	2010	Questionnaire	To help research teams to evaluate dimensions of quality service user involvement in the contexts they are working within
ReseArch with Patient and Public invOlvement: a RealisT evaluation (RAPPORT)	Wilson, Patricia, et al	UK	2015	Log sheet, and Survey	To track the impact of public involvement in research from project inception through to completion or, at a minimum, for complete stages of the research process (design, recruitment, data collection, analysis, dissemination), and to identify the desired outputs and outcomes of public involvement in research from multiple stakeholder perspectives (eg, members of the public, researchers, research managers)
Rifkin spider‐gram	Rifkin, Susan B., Frits Muller, and Wolfgang Bichmann	UK	1988	Spider‐gram	To assess 5 factors influencing community participation in health‐care programmes (needs assessment, leadership, organization, resource mobilization and management). The tools can be used to compare the same programme at a different point in time, to compare observations by different evaluators and/or to compare perceptions of different participants in the same programmes
Scorecard for evaluating engagement	Ontario's Local Health Integration Networks	Canada	2009	Scorecard	To measure 5 consecutive goals necessary to realize a culture of engagement: (i) value public input, (ii) clarity of purpose, (iii) well‐defined roles, (iv) accountability and (v) responsiveness and good communication
Scoresheet for the Tangible Effects of Patient Participation (STEPP)	Kreindler, Sara A., and Ashley Struthers	Canada	2016	Scoresheet	To assess the organizational impact of patient involvement
Survey of Lay members of research ethics committees	Simons, L., G. Wren, and S. Buckland.	UK	2009	Survey	To find out about the range of contributions that lay members are able to provide on Research Ethics Committees
Survey on consumers’ involvement in NHS research	Barber, Rosemary, Jonathan D. Boote, and Cindy L. Cooper.	UK	2007	Survey	To investigate how far and in what way consumers are involved in NHS research
The Participation Toolkit	Scottish Health Council	UK	2014	Checklists, Questionnaire, and Evaluation templates	To evaluate involvement projects and to track progress; to promote good practice and assure staff‐led Patient Focus and Public Involvement work; ensure that learning points and actions are identified and implemented or take forward appropriately; plan, check and/or audit actions for evaluation findings; and improve practices of involvement
Well Connected—a self‐assessment tool on community involvement	South, Jane, Pat Fairfax, and Eleanor Green	UK	2005	Spider‐web	To assess progress and identify areas for improvement on community involvement based on 6 dimensions: diversity, procedures, communication, staff support, opportunities, and resources

Description of included evaluation tools (name, authors, country, year, type and objectives). “Questionnaire” defined as a set of written questions used for collecting information; “Survey” is a set of questions used to aggregate data for statistical analysis; “Scale” is used to measure or order entities with respect to quantitative attributes of traits; “Index” is a compound measure that aggregated multiple indicators in order to summarize and rank specific observations.

### 4.2 Assessment of the tools

Table 3 provides a summary of the evaluation tools’ scores for each of the 4 assessment domains. Assessment scores for each individual assessment criteria are included in Supplementary Document 3.

**Table 3:** Summary assessment scores for all included evaluation tools

	Tools scoring “yes” (n)	Tools scoring “no” (n)	Tools unable to assess (n)	Total score %
Scientific rigour				
Based on literature review	3	20	4	11.1
Based on expertise of key stakeholders	23	0	4	85.2
Based on conceptual/theoretical framework	17	7	3	63.0
Tested for validity	13	6	8	48.1
Tested for reliability	2	18	7	7.4
Patient and public perspective				
Involvement in tool's development	16	3	8	59.3
Involvement in tool's data collection	20	3	4	74.1
Involvement in reporting of results	5	17	5	18.5
Evaluates patient/public engagement activities	25	2	0	92.6
Captures influence of patients and the public	15	10	2	55.6
Comprehensiveness				
Documents the context of engagement	22	5	0	81.5
Documents the process of engagement	20	7	0	74.1
Documents the outcomes of engagement	15	11	1	55.6
Allows monitoring at multiple moments	11	11	5	40.7
Includes open and closed questions	12	11	4	44.4
Usability				
Purpose of the tool is stated	27	0	0	100.0
Tool is freely available	20	3	4	74.1
Tool is available in applicable format	16	10	1	59.3
Tool is easy to read (7th grade level)	3	15	9	11.1
Tool includes instructions for use	14	5	8	51.9

On average, the evaluation tools scored lowest in the scientific rigour domain. The tools were mostly developed based on stakeholder expertise and experience. Only a small number of tools (11.1%) were informed by a review of literature on patient and public engagement in at least 2 databases and 63% were grounded in a specific theoretical or conceptual framework. Reliability testing was rare (7.4% of tools).

Patients and members of the public were involved in the tools’ development more than half the time (59%), but mostly in the piloting stage. Most tools (74.1%) were designed to collect information from patients and the public. However, very few instruments measured the perspectives of patients or members of the public in relation to those of their engagement partners (eg, researchers, clinicians and managers). Only 18.5% of tools were explicitly designed with the objective of reporting back evaluation results to patients and the public.

Five of the tools covered all assessment criteria for comprehensiveness: 2 covering health system decision making and 3 covering research. The outcomes of patient and public engagement were least often evaluated (55.6% of tools), in contrast with the engagement process (74.1%) and context (81.5%). For those tools seeking to evaluate the outcomes of patient and public engagement, the most common focus was on perceived, self‐reported impacts, as opposed to observed impacts by external evaluators.

Two of the tools covered all assessment criteria for usability. All tools described the purpose of the instrument, and the majority (74.1%) were available free of charge. The most important usability issue identified related to readability, with only 11.1% of tools requiring a 7th‐grade or lower reading level.

## 5 DISCUSSION

This systematic review documents a recent growth in patient and public engagement evaluation tools development. We identified 27 evaluation tools for engagement in research and health system decision making, most of which were developed in the last decade. These findings suggest that engagement evaluation activity is increasing around the globe and provide an important basis for future evaluation work.

One of this review major contributions is that it not only identifies existing evaluation tools for patient and public engagement, but it also systematically assesses their main strengths, weaknesses and characteristics using predetermined criteria codeveloped with key stakeholders, thus complementing related work in the area.^28^ The goal of our assessment grid was not to provide an overall quality score, but aimed to guide user's selection of tools to fit their own evaluation needs. As such, our assessment grid cannot be used to “rank” evaluation tools or to identify the “best” tool, but helps identify the strengths and weaknesses of each.

A number of potential weaknesses were identified regarding evaluation tools’ development process. First, scientifically rigorous methods must be used to develop evaluation tools, including more frequent psychometric testing and validation studies.^29^ The fact that only a small number (11%) of instruments are informed by a literature review in at least 2 databases is disconcerting, pointing to a potential duplication of effort (new tools being created because existing ones are unknown) and misalignment with key dimensions of engagement documented in the scientific literature. Secondly, efforts must be made to address the lack of an explicit conceptual framework in most tools, which is significant given the importance of linking empirical evaluation with an explicit theoretical foundation.^6^, ^30^, ^31^ Lastly, the high level of literacy required to understand most instruments should be addressed, particularly because patients and members of the public are the target users of most evaluation tools and because engagement with vulnerable populations is a frequent concern.^32^

In line with the ethos of participation, key stakeholders are often engaged in the development of evaluation tools for engagement. However, the involvement of patients and the public has largely been limited to the data collection stage and rarely extended to the design of evaluation instruments or the reporting of evaluation results.

The predominance of context and process evaluation instruments is surprising, given the frequent calls for greater evaluation of the impact of engagement outcomes.^33^ Evaluating the context and process of engagement is consistent with the objective of developmental and formative evaluation as a means of improving engagement practices.^7^, ^12^, ^34^, ^35^ The number of evaluation tools measuring “perceived self‐reported impacts” as the main measure for outcomes suggests a need for new evaluation tools based on observable impacts.^36^

### 5.1 Strengths and limitations of the review

The main strengths of this review are its comprehensive search strategy and rigorous appraisal of existing tools based on predetermined criteria codeveloped with a broad group of evaluation users. To limit the possibility of omitting relevant material, the search strategy was adapted for each type of database with an information specialist.

Two specific issues may have limited our ability to identify all relevant tools. First, the poor indexing of evaluation tools in some search engines may have contributed to the relatively low number of tools identified. In a further update of this review, additional search engines could be considered to identify additional material. Second, the fact that practitioners rarely publish their evaluation tools—often because of space limitations and journal editors’ publication policies—may also have limited the identification of relevant tools. Many tools have been developed for a single project or for internal use by organizations without being made publicly available. These limitations could be addressed through broader use of publication reporting guidelines for patient and public engagement research.^14^, ^37^ Evaluators and journal editors should pay special attention to publishing evaluation instruments, and properly index them to facilitate the development and use of common instruments across studies.

Furthermore, a number of assessment criteria could not be fully assessed due to a lack of published information. It is possible that certain assessment criteria were applied but not reported by tools’ developers (eg, literature review was performed but not reported), thus reducing the assessment score of the tool. The readability level of about a third of the tools could not be assessed because the complete tool was unavailable.

### 5.2 Implications for practice and research

Patient and public engagement practitioners have access to a broad set of evaluation tools, most of which have been designed to help improve the quality of engagement activities. When developing new instruments or refining existing evaluation tools, particular attention should be given to literature review; alignment with engagement frameworks and theories; readability; impact assessment; psychometric testing; and involvement of patients and the public in evaluation design and reporting.

Most tools identified are targeted instruments evaluating a precise dimension of patient and public engagement, usually concentrating on context, process and perceived self‐reported impact. Future developments could complement existing instruments with more comprehensive evaluation tools that can be used across multiple projects, with evaluation tools’ using a dyadic approach to data collection (eg, assessing engagement from the perspective of patients and their research partners), as well as instruments measuring observable engagement impacts by external evaluators.

The tools identified in this review were assembled and disseminated by means of an open‐access repository of evaluation instruments (http://www.ceppp.ca/en/our-projects/evaluation-toolkit/), thus strengthening engagement practitioners’ capacity for evaluation and reducing duplication of efforts when appropriate instruments already exist. Given the rapid growth of the engagement evaluation field, it is possible that relevant evaluation tools are under development or that existing tools are being refined: an update of this review is recommended in a few years.

## 6 CONCLUSION

A growing set of tools is available for the evaluation of patient and public engagement in research and health system decision making. Knowledge of the tools’ specific strengths and weaknesses can guide practitioners in choosing an appropriate instrument for their evaluation needs. Practitioners developing new tools should place greater emphasis on scientific rigour, the involvement of patients and the public in evaluation design and reporting, and the readability of evaluation instruments. The identification, appraisal and dissemination of existing evaluation tools in an open‐access online repository constitute an important contribution of this review in strengthening collective capacity for evaluating patient and public engagement.
