# Supplementary material for: Patient and public engagement in research and health system decision making: A systematic review of evaluation tools
Source: Health Expect. 2018 Jul 30;21(6):1075–84. doi: 10.1111/hex.12804 (PMC6250878; doi:10.1111/hex.12804)
Supplement: Supplementary file 1 [file HEX-21-1075-s001.pdf]

Supplementary data #1: Assessment grid

Two members of the research team will independently appraise the evaluation tool using the following assessment grid. Discrepancies between the two reviewers will be resolved by consensus.

We provide a five-star rating for each of the following four criteria:

- **Scientific Rigour:** Was the development of the evaluation tool scientifically rigorous and based on existing evidence on patient and public engagement?
- **Patient and Public Perspective:** Does the evaluation tool take into account the views of patients and the public (both in its development and use)?
- **Comprehensiveness:** Is the tool comprehensive in evaluating the context, process, outcomes and impacts of patient and public engagement?
- **Usability:** Is the evaluation tool easy to use?

Each criterion has 5 question-items. We give 1 point per item if we answer YES, 0 if we answer NO or CAN NOT ANSWER.

SCIENTIFIC RIGOUR

- Is the evaluation tool based on a comprehensive literature review on patient and public engagement research?
  Note: To answer YES, at least two electronic sources should have been searched. The evaluation tool must include years and databases used (e.g., Central, EMBASE, and MEDLINE).
  ☐ Yes
  ☐ No
  ☐ Can't answer

- Is the evaluation tool based on the experience/expertise of key stakeholders?
  Note: To answer YES, it must be explicitly stated if key stakeholders (e.g., researchers, patients, clinicians, policy makers) have been consulted in the process of developing the evaluation tool.
  ☐ Yes
  ☐ No
  ☐ Can't answer

- Is the evaluation tool based on a conceptual/theoretical framework of patient and public engagement?
  Note: To answer YES, the framework must provide clear definition of the concept of patient and public engagement and describe how it is operationalized.
  ☐ Yes
  ☐ No
  ☐ Can't answer

- Was the evaluation tool tested for validity (i.e., the tool evaluates what it is purported to evaluate)?
  Note: To answer YES, the evaluation tool must have been tested, at a minimum, for face validity and/or content validity by key stakeholders (e.g., researchers, patients, clinicians, policy makers).
  ☐ Yes
  ☐ No
  ☐ Can't answer

- Was the evaluation tool tested for reliability (i.e., the tool produces stable and consistent results)?
  Note: To answer YES, the evaluation tool must have been tested at least for intra-rater reliability.
  ☐ Yes
  ☐ No
  ☐ Can't answer

PATIENTS AND PUBLIC PERSPECTIVE

- Were patients and/or the public involved in the development of the evaluation tool?
  Note: To answer YES, patients and/or the public must have at least been consulted during the development/piloting of the evaluation tool.
  ☐ Yes
  ☐ No
  ☐ Can't answer

- Is the tool designed to be completed by patients and/or members of the public (self-administered)?
  Note: To answer YES, patients and/or the public must be explicitly identified as the target users of the tool.
  ☐ Yes
  ☐ No
  ☐ Can't answer

- Does the tool explicitly state that the evaluation results must be reported back to patients and the public?
  Note: To answer YES, there must be an explicit statement that the evaluation results are intended to be communicated to the patients and/or the public.
  ☐ Yes
  ☐ No
  ☐ Can't answer

- Was the tool specifically designed to evaluate patient and public engagement

activities?
Note: To answer YES, there must be a clear statement that the tool was designed to evaluate engagement activities, which refer to involvement, collaboration or empowerment on the IAP2 spectrum.
 Yes
 No
 Can't answer

- Does the tool captures the influence of patients and the public? (e.g., on the engagement process, on the final decisions, etc)
  Note: To answer YES, the tool must ask at least one question about the participants' perception of the patient/public member's contribution.
   Yes
   No
   Can't answer

COMPREHENSIVENESS

- Does the tool document the context of engagement?
  Note: To answer YES, the tool must at least ask one question about the internal and/or external context of patient and public engagement (e.g.,... the nature of the issue, ...).
   Yes
   No
   Can't answer

- Does the tool document the process of engagement?
  Note: To answer YES, the tool must have at least one question about how patients and/or the public are engaged.
   Yes
   No
   Can't answer

- Does the tool document the outcome/impact of engagement?
  Note: To answer YES, the tool must have at least one question about the perceived outcomes/impact of patient or public engagement.
   Yes
   No
   Can't answer

- Does the tool monitor the engagement process at multiple moments?
  Note: To answer YES, the tool is explicitly designed to be used more than once during the project.
   Yes

☐ No
☐ Can’t answer

- Does the tool consist of a set of open and closed questions?
  Note: To answer YES, the tool must consist of a combination of both types of questions (one of each at least).
  ☐ Yes
  ☐ No
  ☐ Can’t answer

USABILITY

- Is the purpose of the evaluation tool stated?
  Note: To answer YES, the purpose of the evaluation tool must be explicitly stated.
  ☐ Yes
  ☐ No
  ☐ Can’t answer

- Is the evaluation tool freely available?
  Note: To answer YES, the evaluation tool should be freely accessible through an open access journal or publicly available on the Web.
  ☐ Yes
  ☐ No
  ☐ Can’t answer

- Is the evaluation available in a applicable format?
  Note: To answer YES, the evaluation tool should be accessible in its complete form and ready to be used.
  ☐ Yes
  ☐ No
  ☐ Can’t answer

- Is the evaluation tool easy to read and understand?
  Note: To answer YES, the evaluation tool must score between 70 and 100 at the Flesch reading-ease test (someone who has at least 7th grade reading capacity level).
  ☐ Yes
  ☐ No
  ☐ Can’t answer

- Is the tool accompanied by instructions for use?
  Note: To answer YES, instructions must be provided about how to use the tool.
  ☐ Yes
  ☐ No
  ☐ Can’t answer
