# Supplementary material for: Patient and public engagement in research and health system decision making: A systematic review of evaluation tools
Source: Health Expect. 2018 Jul 30;21(6):1075–84. doi: 10.1111/hex.12804 (PMC6250878; doi:10.1111/hex.12804)
Supplement: Supplementary file 2 [file HEX-21-1075-s002.pdf]

Supplementary table #1: Evaluation tool's individual assessment scores

	Scientific rigour						Patient and public perspective				Comprehensiveness						Usability			
	Litterature review	Stakeholder expertise	Theoretical	Validity	Reliability	Development	Collection	Reporting	Engagement	Influence	Context	Process	Outcome	Multiple	Open/Close	Purpose	Free	Format	Readibility	Instructions
A Scorecard for evaluating engagement	0	1	0	0	0	1	NA	1	1	1	1	1	1	NA	0	1	1	0	0	0
Measuring Organisational Readiness for patient Engagement (MORE)	0	1	0	1	0	1	NA	NA	1	0	1	0	0	NA	NA	1		0	NA	NA
PEI Engagement Toolkit	0	1	1	NA	NA	0	1	0	1	1	1	1	1	NA	1	1	1	1	0	1
The Involvement Portfolio	0	1	0	NA	NA	1	1	NA	1	0	1	1	0	1	0	1	1	1	0	1
Checklist for attitudes for patients and families as advisors	NA	NA	NA	NA	NA	NA	0	0	0	0	1	0	0		0	1	1	1	0	1
Survey on consumers' involvement in NHS research	0	1	1	1	0	1	1	1	1	1	0	1	0	0	NA	1		0	NA	NA
A resource toolkit for engaging patient and families at the planning table	0	1	0	NA	NA	NA	1	0	1	0	1	1	0	1	1	1	1	1	0	1
Partnership Assessment In community-based Research (PAIR)	1	1	1	0	0	1	1	NA	1	NA	1	1	NA	1	NA	1		0	NA	NA
Evaluating the participatory process in a community-based heart health project	NA	NA	NA	NA	NA	NA	NA	NA	1	NA	0	1	1	0	1	1	NA	NA	NA	1
Well Connected – a self-assessment tool on community involvement	1	1	1	1	0	1		1	1	0	1	1	0	1	NA	1		0		1
PCORI Engagement Activity Inventory (netENACT and We-ENACT)	NA	NA	1	NA	NA	NA	1	0	1	1	1	1	1	1	1	1	1	1	1	0
Public Involvement Impact Assessment Framework (PiiAF)	NA	1	1	0	0		1	NA	1	1	1	1	1		1	1	1	1	0	1
Public and Patient Engagement Evaluation Tool (PPEET)	1	1	1	1	0	1	1	0	1	1	1	1	1	1	1	1	1	1	0	1
Rifkin spider-gram	0	1	1	NA	NA	NA	1	0	0	0	1	1	0	1	0	1	1	0	NA	NA
Quality Involvement Questionnaire	0	1	1	NA	NA	NA	1	1	1	1	1	1	0	1	0	1	1	1	1	0
Scoresheet for the Tangible Effects of Patient Participation (STEPP)	0	1	1	1	1	1	1	NA	1	1	0	0	1	0	0	1	0	1	0	1
ReseArch with Patient and Public invOlvement: a RealisT evaluation (RAPPORT)	0	1	1	0	0	0	1	1	1	1	1	1	1	1	1	1	1	1	0	1
Organisational Self-Assessment and Planning (OSAP) Tool	0	1	1	1	0	1	1	0	1	0	1	1	1	1	1	1	1	1	0	1
Kroutil Checklist	0	1	1	1	0	0	0	0	1	0	1	0	0	0	0	1	0	0	NA	NA
The Participation Toolkit	0	1	0	0	0	1	1	0	1	1	1	1	1	0	1	1	1	1	1	1
Engaging patients as partners in practice improvement	0	1	1	1	0	1	0	0	1	1	1	0	1	0	0	1	1	1	0	0
Survey of Lay members of research ethics committees	0	1	NA	1	0	1	1	0	1	1	1	0	1	0	1	1	1	1	0	1
Health Democracy Index	0	NA	0	1	1		1	0	1	1	1	0	1	0	0	1	1	0	NA	NA
Community Engagement in Research Index (CERI)	0	1	1	1	0	1	1	0	1	0	0	1	0	0	0	1	1	0	NA	NA
Community engagement and participation in research measure	0	1	1	1	0	1	1	0	1	1	1	1	1	0	0	1	1	0	NA	NA
Patients as Partners in Research Surveys	0	1	0	1	0	1	1	0	1	1	1	1	1	1	1	1	1	1	0	1
An Evaluation of In-Person and Online Engagement in Central Newfoundland	0	1	1	0	0	1	1	0	1	0	0	1	0	0	1	1	1	1	0	0

1=yes
0=no
NA=unable to answer
